# Genomic and clinical epidemiology of SARS-CoV-2 in Lebanon: a prospective multicenter study 2020–2024

**DOI:** 10.1186/s12879-026-12635-w

**Published:** 2026-01-28

**Authors:** Fatima Dakroub, Celina F. Boutros, Diego Teixeira, Habib Alkalamouni, Nagham Hamzah, Nancy Hourani, Amani Haddara, Samar Dalle, Dima Khreis, Elsy Tawil, Silma Baasiri, Zeinab El Zein, Mayse Naser, Rawan Korman, Maher Sraj, Yara Salameh, Sarah Merhi, Kawthar Faour, Nadim Tfaily, Stephanie Damaj, Mohammad Taleb, Soha Ghanem, Ghenwa Dakdouki, Rouba Shaker, Imad Chokr, David Breish, Maria Karam, Nada M. Melhem, Antoine Abou Fayad, Hassan Zaraket, Ewan M. Harrison, Ghassan M. Matar, Ghassan S. Dbaibo

**Affiliations:** 1 Center for Infectious Diseases Research (CIDR) and WHO Collaborating Center for Reference and Research on Bacterial Pathogens, American University of Beirut, Beirut, Lebanon; 2 Parasites and Microbes Programme, Wellcome Sanger Institute, Hinxton, UK; 3 Department of Pediatrics and Adolescent Medicine, American University of Beirut, Beirut, Lebanon; 4 Department of Experimental Pathology, Immunology, and Microbiology, Faculty of Medicine, American University of Beirut, Beirut, Lebanon; 5 Department of Pediatrics, Makassed General Hospital, Achrafieh, Beirut, Lebanon; 6 Department of Pediatrics, Hammoud Hospital University Medical Center, Saida, Lebanon; 7 Department of Pediatrics, New Mazloum Hospital, Tripoli, North, Lebanon; 8 Department of Pediatrics, Rafic Hariri University Hospital, Beirut, Lebanon; 9 Department of Pediatrics, Bekaa Hospital, Bekaa, Lebanon; 10 Department of Pediatrics, Keserwan Medical Center, Jounieh, Mount Lebanon Lebanon; 11 Medical Laboratory Sciences Program, Division of Health Professions, Faculty of Health Sciences, American University of Beirut, Beirut, Lebanon; 12 Department of Medicine, University of Cambridge, Cambridge, UK; 13 Center for Infectious Diseases Research, Department of Pediatrics and Adolescent Medicine, American University of Beirut Medical Center, Beirut, 1107 Lebanon; 14 Center for Infectious Diseases Research, Department of Experimental Pathology, Immunology, and Microbiology, Faculty of Medicine, American University of Beirut, Beirut, 1107 Lebanon

**Keywords:** COVID-19, SARS-CoV-2, Pneumonia, Epidemiology, Whole genome sequencing, Clinical characteristics

## Abstract

**Background:**

Globally, the trajectory of COVID-19 has been shaped by viral evolution, widespread vaccination and immunity from prior infections. We assessed the epidemiological and clinical patterns of COVID-19 in Lebanon between 2020 and 2024, identified the predominant SARS-CoV-2 clades and evaluated risk factors for COVID-19 associated mortality.

**Methods:**

This multicenter prospective study enrolled 1302 patients hospitalized with COVID-19 in Lebanon between November 2020 and October 2024. Multivariate logistic regression was used to determine predictors of COVID-19 associated mortality. Whole genome sequencing (WGS) was utilized to investigate the genomic epidemiology of SARS-CoV-2 and infer viral interactions between Lebanon and other countries. Multiple sequence alignment and phylogenetic analysis were conducted using the augur pipeline.

**Results:**

A progressive and significant reduction in severe outcomes, including pneumonia and mortality was observed throughout the study period. Pneumonia (AOR, 6.714; CI, 4.140–10.888; *p* < 0.0001) and age ≥ 60 years (AOR, 6.051; CI, 2.190–16.723; *p* = 0.001) were identified as independent predictors of COVID-19 mortality. Moreover, receiving 3 doses of a COVID-19 vaccine significantly reduced the odds of mortality (AOR, 0.229; CI, 0.108–0.486; *p* < 0.0001). Genomic analysis revealed multiple introductions of the same SARS-CoV-2 clades into Lebanon, which seeded local transmission chains.

**Conclusions:**

The transition of COVID-19 from pandemic to endemic in Lebanon was associated with reduced disease severity. Vaccination remains essential, particularly in older adult patients who are at high risk of mortality. Moreover, early diagnosis and management of pneumonia are crucial, given its association with COVID-19 mortality. Furthermore, WGS has proven valuable in tracking the local evolution of SARS-CoV-2 and its impact on clinical outcomes.

**Supplementary Information:**

The online version contains supplementary material available at 10.1186/s12879-026-12635-w.

## Background

In December 2019, SARS-CoV-2 emerged in Wuhan, China and quickly became a global public health emergency with significant morbidity and mortality [1, 2]. It has become a major etiologic agent of severe acute respiratory infection (SARI) and is associated with severe manifestations such as pneumonia and acute respiratory distress syndrome (ARDS) [3]. Tracking viral evolution over time and its impact on the clinical progression of coronavirus disease 2019 (COVID-19) is important for proper understanding and management of the disease. Early in the pandemic, several risk factors were identified for COVID-19 severity and mortality, including older age and comorbidities such as diabetes and cardiovascular disease (CVD) [4, 5]. Identifying these risk factors helped shape public health policies, with high-risk individuals prioritized during vaccination campaigns. However, the trajectory of the COVID-19 pandemic has shifted over time, altering the clinical presentation, severity, and outcomes of the disease [5, 6]. This has been attributed to factors such as viral evolution, widespread vaccination and immunity from prior infections.

The pathogenicity of SARS-CoV-2 has shifted through rapid evolution and the accumulation of multiple mutations, which may directly impact clinical outcomes [7]. For example, the Alpha (B.1.1.7) variant was found to be associated with increased COVID-19 severity among hospitalized women but not men [8]. Patients infected with the Delta variant were shown to have higher levels of inflammatory markers than those infected with previous variants [9]. The Omicron variant, which emerged in November 2021 as the dominant strain globally, was associated with milder COVID-19 and fewer hospitalizations [10–12]. These reports underscore the importance of genomic surveillance to track SARS-CoV-2 variants and assess their clinical implications. In Lebanon, we previously reported Omicron as the dominant variant among healthcare workers in December 2021 and January 2022 [13]. However, there remains a paucity of data on the genomic epidemiology and clinical burden of SARS-CoV-2 in Lebanon.

Immunity from infections and widespread vaccination have additionally contributed to the transition of COVID-19 from a pandemic to an endemic disease. These factors altered the clinical course of COVID-19, including the spectrum and severity of acute and prolonged symptoms [14, 15]. The primary SARS-CoV-2 vaccines used during the pandemic were mRNA-based, and their safety and effectiveness have been well documented [16, 17]. The Pfizer-BioNTech and Moderna mRNA vaccines exhibit high efficacy rates against symptomatic infection, exceeding 90% [18]. Beyond licensed vaccines, circular RNAs [19] and intranasal vaccines [20] represent promising future vaccine candidates against SARS-CoV-2. However, vaccination strategies should generally account for evolving viral strains and population characteristics [21]. Given these factors, assessing the local epidemiological and clinical patterns of COVID-19 in Lebanon, where population-level immunity and variant exposure may differ from global trends, is essential.

This study aimed to compare the clinical characteristics of COVID-19 across four consecutive years in Lebanon to enhance our understanding of disease dynamics. Moreover, we aimed to identify risk factors associated with COVID-19 mortality. We examined age-related differences in disease presentation and compared SARI cases associated with SARS-CoV-2 with those who tested negative. Finally, genomic analysis was conducted to define the clinical findings within the context of SARS-CoV-2 evolution.

## Materials and methods

### Study design

We conducted a multicenter observational study across six hospitals in Lebanon, representing the northern, central, southern and eastern regions. The study sites are summarized in Table S1. Samples were collected as part of the Global Influenza Hospital Surveillance Network (GIHSN), a hospital-based surveillance program designed to collect epidemiological data on respiratory viruses. The GIHSN study is approved by the AUB institutional review board and informed written consent is obtained from all study participants or from their legal representatives when participants are unable to provide consent.

Patients were eligible for inclusion if they had been hospitalized within the previous 72 hours, with a minimum of one overnight stay. Prior to 2021/2022, children under five years of age were included if symptom onset occurred within seven days before hospital admission. Patients aged five years and older were eligible if they met the European Centre for Disease Prevention and Control (ECDC)-modified case definition for influenza-like illness (ILI) [22]. The latter required at least one systemic symptom and at least one respiratory symptom within the previous seven days. Since 2021/2022, the World Health Organization (WHO) Extended SARI case definition was adopted, which includes any acute respiratory infection with cough and symptom onset within the past 10 days that requires hospitalization. Patients who did not consent to participate and those with a history of hospitalization within the previous 30 days were excluded from the study. Repeated infections were included only if at least one month had elapsed between episodes. Each eligible episode was documented and analyzed independently. Sample Processing and analyses were conducted at the Center for Infectious Diseases Research (CIDR), which is part of the GIHSN network. Additionally, a subset of samples was tested for SARS-CoV-2 at the participating hospitals.

### Sample and data collection

Demographic and clinical data were collected by trained physicians and nurses via personal interviews with patients or their parents/legal guardians and recorded in a structured case report (CRF). The CRF was based on questionnaires recommended for each respiratory season by the GIHSN network [23]. Data extracted from electronic medical records were additionally incorporated into the CRF. Clinical data included comorbidities, symptoms and their duration, prior and ongoing use of antiviral or antibiotic medications, history of influenza and COVID-19 vaccinations, complications, length of hospitalization and outcomes. Immunocompromised status was defined as having active malignancy, inborn errors of immunity, a history of solid organ transplantation or any autoimmune disease requiring immunosuppressive therapy. Patients were followed up until in-hospital death or discharge.

For patients who had previously provided nasopharyngeal swabs for viral testing at the hospital, any residual sample was retrieved and an additional oropharyngeal swab was requested. If no sample was available, nasopharyngeal and oropharyngeal swabs were collected from participants aged 14 years and older. For children aged < 14 years, only nasal swabs were collected. The specimens were kept at −20 °C and transported within 72 hours to the CIDR lab for processing.

### RNA extraction and multiplex PCR

Viral RNA was extracted from 250 µl per sample using the High Pure Viral RNA Kit (Roche, Switzerland) according to the manufacturer’s instructions. RNA was converted to cDNA and amplified in a multiplex reaction containing four sets of primers and probes for the detection of influenza A, influenza B, respiratory syncytial virus (RSV) and SARS-CoV-2. The Reliance One-Step Multiplex Super mix kit (Bio-Rad, California, US) was used for PCR amplification. Each run included two negative controls consisting of a template-free reaction tube and a mock extraction control prepared with nuclease-free water. Samples with a cycle threshold (CT) below 38 were considered positive. A total of 1061 out of the 1302 (81.5%) SARS-CoV-2 cases in this study were confirmed via multiplex PCR. The COVID-19 diagnoses (documentation of a positive PCR or rapid antigen test) for the remaining 241 cases (18.5%), for which excess samples were not available, were obtained from medical records. SARS-CoV-2 samples with a sufficient viral load, defined as a CT < 28, were selected for whole genome sequencing (WGS).

### Whole genome sequencing and data analysis

A total of 166 SARS-CoV-2 viruses were sequenced using the Oxford Nanopore Minion (*n* = 101) or Illumina MiSeq (*n* = 65) platforms. Of these, a total of 138 genomes passed quality control (QC) and were included in downstream analyses. The Oxford Nanopore Rapid Barcoding Kit 96 and Midnight RT PCR Expansion were used for long-read sequencing. The library was loaded into R9.4.1 or R10.4.1 flow cells (FLO-MIN114) and sequenced using the MinION Mk1C device (Oxford Nanopore Technologies, UK). The Illumina COVIDSeq assay library preparation kit (Illumina, San Diego, CA) was used for short-read sequencing per the manufacturer’s protocol. The MiSeq V2 Reagent Kit was used for sequencing the pooled DNA library (Illumina, San Diego, CA).

Consensus sequences from Illumina data were generated using the viral recon pipeline (v1.0.0-rc12) (https://github.com/nf-core/viralrecon) with default parameters and the Illumina amplicon analysis option. Oxford Nanopore data were processed using either the epi2me-labs/wf-artic pipeline (https://github.com/epi2me-labs/wf-artic) or the Nanopore amplicon analysis option in the nf-core/viralrecon pipeline, both implementing the ARTIC workflow. Samples were included in downstream analyses if they had a median sequencing depth above 20x and at least 70% of the genome covered at > 10x. Samples meeting these criteria but lacking a lineage assignment were excluded, as this indicates low coverage at variant/genotyping loci. Consensus sequences were uploaded to Nextclade (https://clades.nextstrain.org/) for mutation calling and lineage assignment.

A maximum likelihood phylogenetic tree was constructed using sequences from 138 genomes from this study and 576 genomes from global circulation. Global SARS-CoV-2 identifiers were obtained from the Nextstrain Global all-time dataset on 11/07/2025. The corresponding sequences and metadata were then downloaded from GISAID and subsampled to exclude clades not found among the study isolates. Two sequences from clade 22D (BA.2.75) were manually added into the final dataset. A time resolved phylogeny inference under discrete geographic reconstruction was performed by the Augur toolkit [24] with MAFFT (v7.526) [25] for sequence alignment, IQ-TREE (v2.3.6) [26] for phylogenetic inference, under the best fitting model of nucleotide substitution as determined by ModelFinder, and TreeTime (v. 0.11.4) [27] to calibrate the inferred phylogenetic tree into time units. The tree topology was exported using ggtree in R (v4.5.0) [28].

Chord diagrams of inferred SARS-CoV-2 genome transitions involving Lebanon were constructed using a dataset retrieved from GISAID. The complete metadata was downloaded on August 19, 2025 and genomes shorter than 26 kb were excluded from further analysis. To ensure a representative phylogeny, one genome per country per lineage per epidemiological week was randomly selected to construct a maximum likelihood phylogenetic tree. A total of 52,775 genomes were initially included, with 16 removed during the regression analysis, resulting in a final tree of 52,759 genomes. For each node in the phylogenetic tree, the traits of the parent nodes were determined using the traits reconstruction file, and this information was applied to investigate the countries interacting with Lebanon. These interactions were visualized via the chordDiagram function from the circlize (v.0.4.16) package in R [29].

A third phylogenetic tree was constructed with the 138 genomes from this study and 85 genomes from Lebanon, retrieved from GISAID. The augur pipeline (version 31.3.0) was used to construct the tree via the IQ-TREE method. The alignment was generated by using Wuhan-Hu-1 as a reference genome (ENA accession number MN908947) and the MAFFT method in the pipeline.

### Statistical analysis

Statistical analysis was performed using GraphPad (v8.4) for Windows (GraphPad Software, La Jolla, California, USA). Categorical variables were analyzed using the chi-square test (X^2^) or Fisher’s exact test. Continuous variables were analyzed using the Mann‒Whitney U test. The normality of the data was tested using the Kolmogorov–Smirnov test. Variables with missing data were excluded from analysis. Therefore, percentages in Table 1 were calculated based on the number of patients with non-missing data, and no imputation was performed.

**Table 1:** Characteristics and clinical outcomes of hospitalized COVID-19 patients across four years in Lebanon

	Year					
	2020/2021 (n = 391)	2021/2022 (n = 358)	2022/2023 (n = 307)	2023/2024 (n = 246)	All patients (n = 1,302)	p-value
Demographics						
Male, n (%)	246 (63%)	199 (56%)	167 (54%)	141 (57%)	753 (58%)	0.0939
Age; years; mean (±SD)	55.55 (19.6)	43.47 (33.1)	50.28 (32.47)	39.9 (34.58)	48.02 (30.42)	< 0.0001
Age < 18 years, n (%)	14 (4%)	120 (34%)	84 (27%)	101 (41%)	319 (25%)	< 0.0001
Age 18–59 years, n (%)	197 (50%)	72 (20%)	54 (18%)	45 (18%)	368 (28%)	< 0.0001
Age ≥ 60 years n (%)	180 (46%)	166 (46%)	169 (55%)	100 (41%)	615 (47%)	0.0069
Clinical Characteristics n (%)						
Chronic Conditions	260 (66%)	221 (62%)	214/306 (70%)	145 (59%)	840/1,301 (65%)	0.0274
Cardiovascular Diseases	172 (44%)	152 (42%)	169/306 (55%)	96 (39%)	589/1,301 (45%)	0.0007
Diabetes	110 (28%)	74 (21%)	80/306 (26%)	48 (20%)	312/1,301 (24%)	0.0252
Immunocompromised	42 (11%)	50 (14%)	43/306 (14%)	27 (11%)	162/1,301 (12%)	0.3896
COVID-19 Vaccination	47/233 (20%)	141/347 (41%)	161/306 (53%)	108/244 (44%)	457/1,130 (40%)	< 0.0001
Vaccine Doses ≥ 3	10/233 (4%)	74/347 (21%)	101/306 (33%)	65/244 (27%)	250/1,130 (22%)	< 0.0001
Outcomes n (%)						
ICU Admission	152/390 (40%)	72 (20%)	84 (27%)	44 (18%)	352/1,301 (27%)	< 0.0001
Oxygen Supplementation	267/354 (75%)	159/355 (45%)	112/306 (37%)	71/244 (29%)	609/1,259 (48%)	< 0.0001
Mechanical Ventilation	69/390 (18%)	27/357 (8%)	29/303 (10%)	9 (4%)	134/1,296 (10%)	< 0.0001
Complications	144/378 (38%)	79/352 (22%)	78/306 (25%)	34 (14%)	335/1,282 (26%)	< 0.0001
Bacterial Co-infections	77/353 (22%)	62/304 (20%)	73/244 (30%)	69/235 (29%)	281/1,136 (25%)	0.0122
Chest X-ray Confirmed Pneumonia*	93 (24%)	57 (16%)	26 (8%)	8 (3%)	184 (14%)	< 0.0001
ARDS	72 (18%)	16 (4%)	14 (5%)	5 (2%)	107 (8%)	< 0.0001
Mortality	75/389 (19%)	29 (8%)	27/306 (8%)	10 (4%)	141/1,299 (11%)	< 0.0001

Abbreviations: ARDS, Acute respiratory distress syndrome; ICU, Intensive care unit

*Radiologically confirmed pneumonia that developed during hospitalization

The Kaplan‒Meier method was used to estimate survival in the total population according to whether they developed pneumonia. Survival was also estimated based on the need for mechanical ventilation. Survival time was calculated from the date of hospital admission and patients who were transferred to other hospitals were not included in the survival analysis. The curves were compared via the log-rank (Mantel‒Cox) test.

Multivariate logistic regression was performed using IBM SPSS Statistics for Windows version 29 (Armonk, NY) to determine the factors associated with COVID-19 mortality. Variables were selected based on clinical relevance and statistical significance in the univariate analysis. Unadjusted odds ratios (UOR), adjusted odds ratios (AOR), and their corresponding 95% confidence intervals (CI) were reported. All tests were two-tailed, and p-values < 0.05 were considered statistically significant.

## Results

### SARS-CoV-2 epidemic trends in Lebanon

**Fig. 1:**
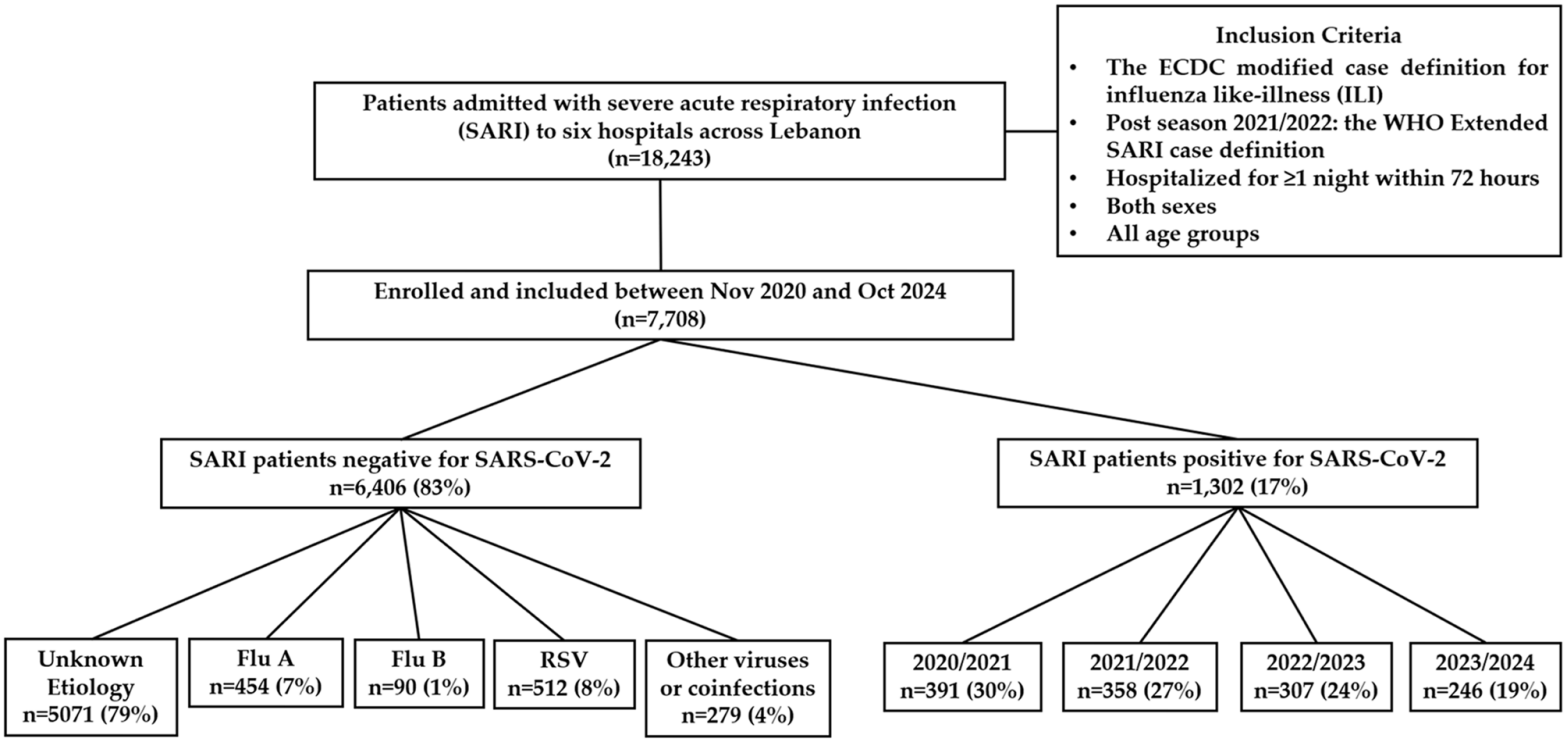
The flowchart of the study

Between November 2020 and October 2024, a total of 7708 patients hospitalized with SARI were prospectively enrolled (Fig. 1). Among these, COVID-19 was confirmed in 1302 patients (17%), while influenza A and B and RSV were identified in 1056 patients (14%). The yearly distribution of COVID-19 cases (*n* = 1,302) was as follows: 391 (30%) in 2020/2021, 358 (27%) in 2021/2022, 307 (24%) in 2022/2023, and 246 (19%) in 2023/2024. A surveillance year was defined as starting in November and ending in the following October.

**Fig. 2:**
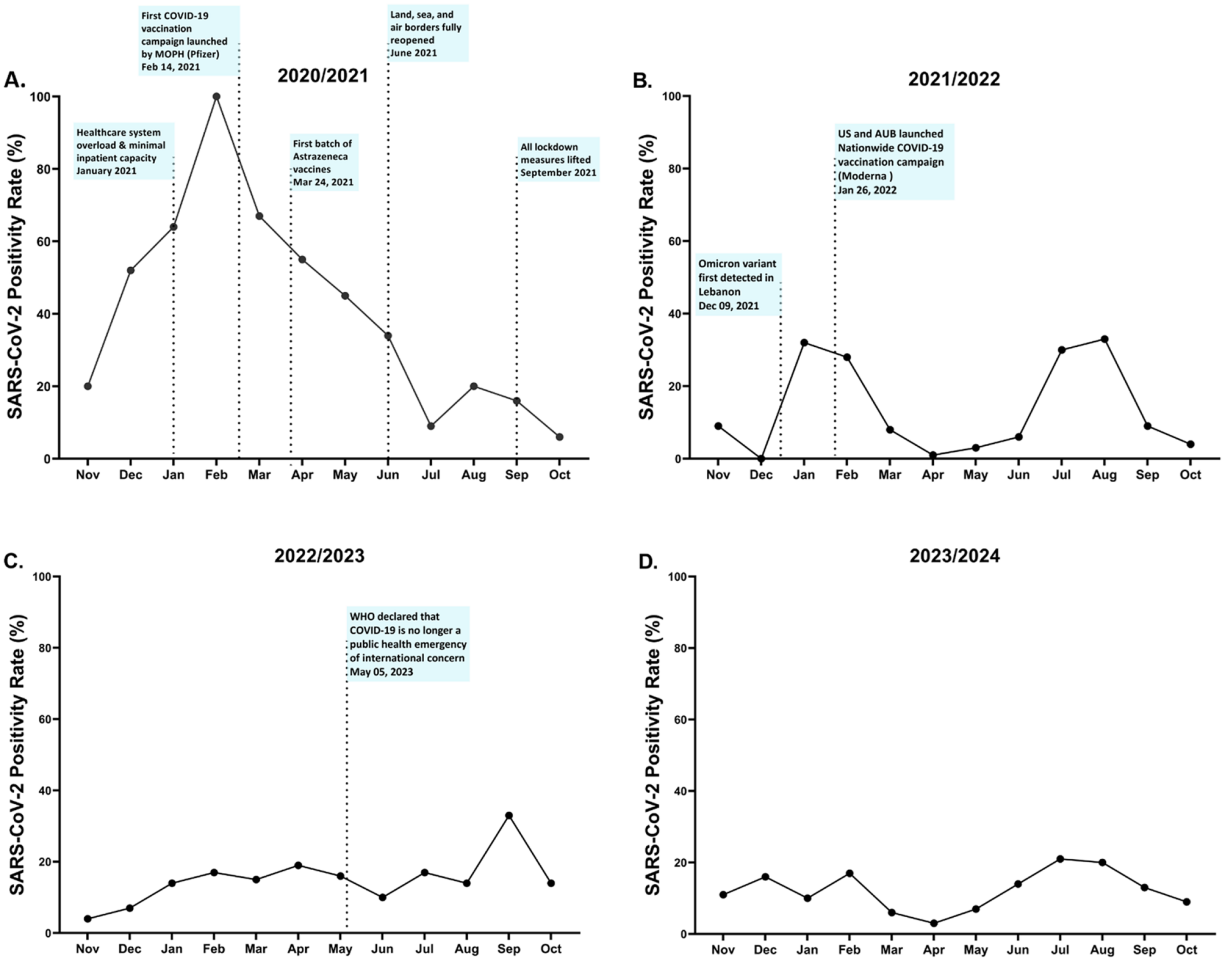
The SARS-CoV-2 positivity rate among hospitalized SARI patients (*n* = 7,708) Lebanon (2020–2024). The key pandemic measures and events, including lockdowns and vaccination campaigns, are shown in the figure according to their date of implementation

In this study the overall SARS-CoV-2 positivity rate was highest in 2020/2021 (37%) and lowest in 2023/2024 (12%) (Figure S1). Over the study period, the largest wave of SARS-CoV-2 infections occurred during the year 2020/2021, spanning from November 2020 to July 2021 (Fig. 2A). In January 2021, the SARS-CoV-2 positivity rate among the SARI patients included in this study reached 64% (*n* = 21/33), coinciding with national reports of healthcare system strain and critical hospital bed shortages. Moreover, the first COVID-19 national vaccination campaign was launched by the Ministry of Public Health (MOPH) in February 2021. In 2021/2022, we observed two distinct waves, with SARS-CoV-2 positivity rates peaking at 31.6% in January 2022 and 33.1% in August 2022 (Fig. 2B). One of the key public health milestones during this year, was the launch of a second nationwide COVID-19 vaccination campaign in January 2022 by the USA and AUB. In contrast to previous years, 2022/2023 and 2023/2024 were characterized by sustained, lower-level SARS-CoV-2 circulation without distinct epidemic peaks (Figs. 2C and 2D).

### Demographic and clinical characteristics

The mean age at hospital admission for all COVID-19 patients included in this study was 48 years (±30.42), with males accounting for 58% of the cohort (Table 1). A total of 319 patients (25%) were aged 18 years or younger, 368 patients (28%) were aged between 18 and 59 years, and 615 patients (47%) were over 60 years. Interestingly, pediatric patients aged < 18 years constituted 61% (*n* = 3,874/6,403) of other SARI cases that were negative for SARS-CoV-2 (Table S2). Most COVID-19 patients had at least one underlying chronic condition (65%), with CVD being the most frequently reported (45%), followed by diabetes (24%) (Table 1). Moreover, immunocompromised patients constituted 12% of the cohort.

Among those with available vaccination data (*n* = 1,130), 40% had received at least one dose of a COVID-19 vaccine. Oxygen supplementation was required in 48% of the patients, and 10% were mechanically ventilated. A total of 352 patients (27%) were admitted to the intensive care unit (ICU). Pneumonia and ARDS occurred in 14 and 8% of patients respectively. The overall in-hospital mortality rate among COVID-19 patients with available mortality data (*n* = 1,299) was 11%. Compared to COVID-19 patients, other SARI patients that were negative for SARS-CoV-2 had significantly lower rates of ICU admission (19%) and mortality (3%) (*p* < 0.0001) (Table S2). The incidence of complications in this group, including ARDS (2%) and pneumonia (7%), was significantly lower than in COVID-19 patients, (*p* < 0.0001).

### Comparison of COVID-19 patient characteristics over time

A progressive reduction in the severe outcomes of COVID-19 was observed over time in Lebanon. ICU admissions decreased significantly from 40% in 2020/2021 to 18% in 2023/2024 (*p* < 0.0001). The proportion of patients requiring oxygen supplementation decreased from 75% to 29% over the same period (*p* < 0.0001). Similarly, mechanical ventilation rates decreased from 18% in 2020/2021 to 4% in 2023/2024 (*p* < 0.0001). The incidence of complications, including ARDS and radiologically confirmed pneumonia, followed a similar decreasing trend. Notably, the prevalence of pneumonia among COVID-19 patients significantly decreased from 24% in 2020/2021 to 3% in 2023/2024, the most recent study period. A significant reduction in mortality was also observed, decreasing from 19% in 2020/2021 to 8% in both 2021/2022 and 2022/2023, and then further declining to 4% in 2023/2024 (*p* < 0.0001).

A notable shift in the age distribution of COVID-19 patients was observed over the study period. In 2020/2021, COVID-19 SARI primarily affected adults, where 50% of patients were aged between 18 and 59 years, 46% were aged ≥60 years, and only 4% were younger than 18 years. By 2023/2024, a significant increase (41%) in pediatric cases was detected. This was accompanied by a significant decrease in the proportion of COVID-19 patients aged between 18 and 59 years to 18% (*p* < 0.0001).

### COVID-19 outcomes by age group

**Fig. 3:**
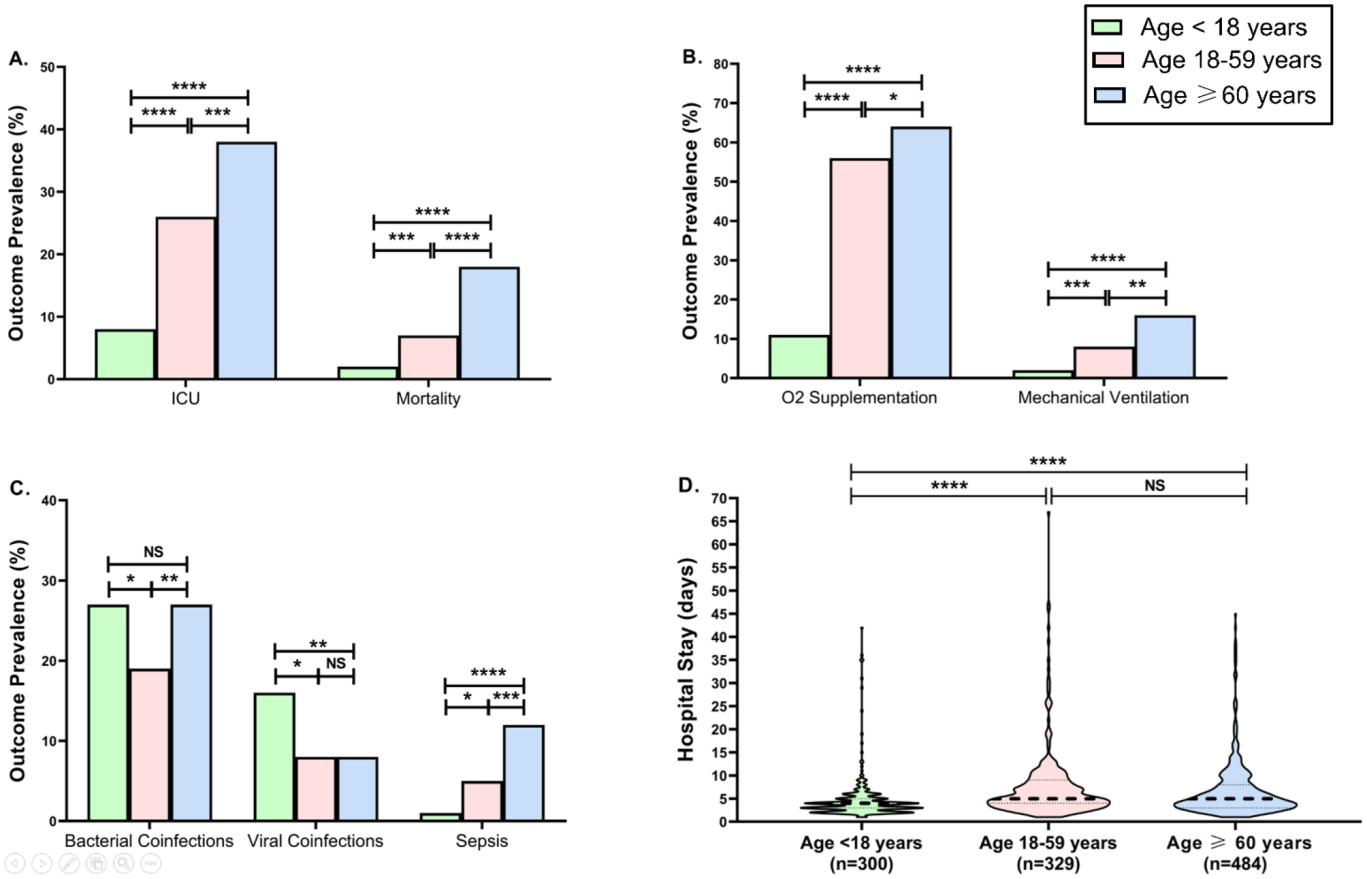
The incidence of mortality, ICU admission, pneumonia (**A**), Oxygen supplementation, mechanical ventilation (**B**), Sepsis and bacterial and viral coinfections (**C**) According to age group. Panel D presents the violin plots of the average hospital stay by age group (**D**). Patients who died or were transferred to other hospitals were excluded from the hospital stay analysis

We examined COVID-19 outcomes across different age groups. Compared with pediatric patients, older adults exhibited significantly higher rates of ICU admission (38% vs 8%, *p* < 0.0001), mortality (18% vs 2%, *p* < 0.0001) and radiologically confirmed pneumonia (19% vs 2.5%, *p* < 0.0001) (Fig. 3A). Moreover, the need for mechanical ventilation was significantly higher among the ≥ 60 years age group (16%) compared to patients aged between 18 and 59 years (8%, *p* = 0.0012) and those younger than 18 years (2%, *p* < 0.0001) (Fig. 3B). Similarly, sepsis was more prevalent in older adults (12%) than in pediatric patients (1%, *p* < 0.0001) and in those aged between 18 and 59 years (5%, *p* = 0.0002). Notably, the incidence of viral coinfections was highest in the < 18 years age group (16%) (Fig. 3C). The incidence of bacterial coinfections was significantly lower in patients aged between 18 and 59 years (19%) than in pediatric (27%, *p* = 0.0220) and older adult patients (27%, *p* = 0.0072). The median length of hospital stay was 4 days (IQR: 3–5) in the pediatric group and 5 days (IQR: 3–8) in the older adult group (*p* < 0.0001) (Fig. 3D).

### Predictors of COVID-19 associated mortality

**Fig. 4:**
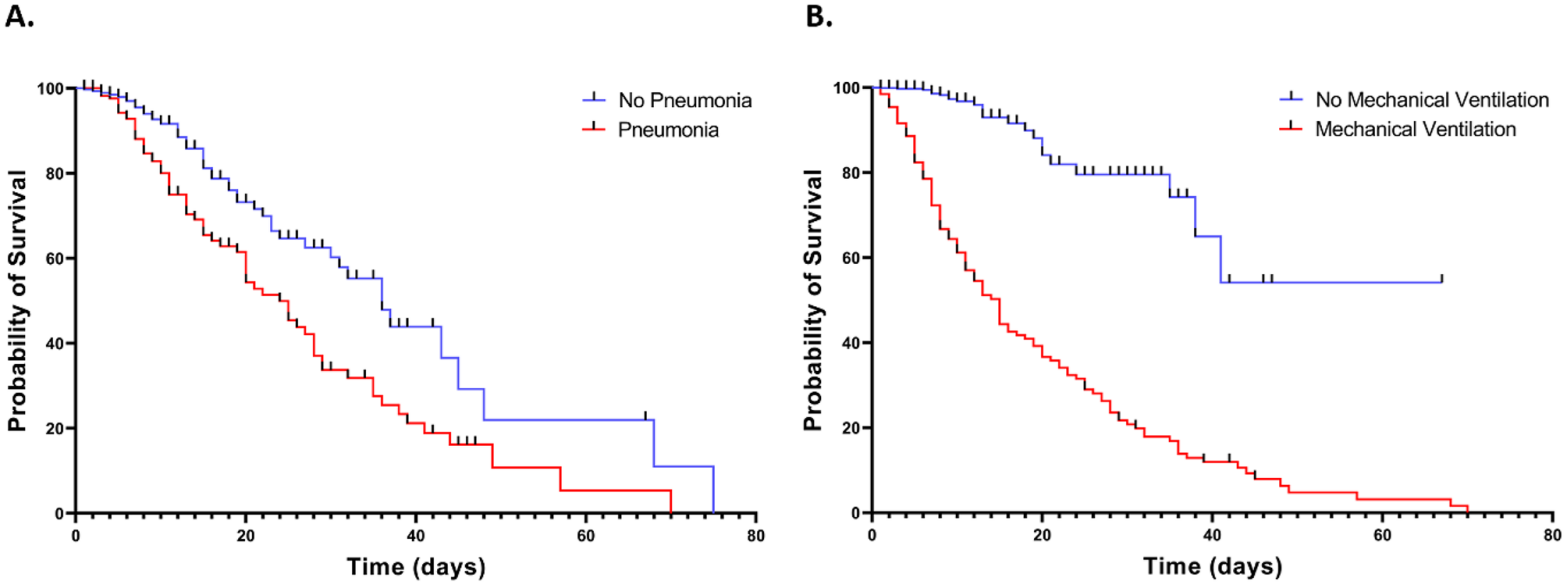
Kaplan–Meier survival plot according to the occurrence of pneumonia during hospitalization (**A**) or the need for mechanical ventilation (**B**). The p-value was < 0.0001 for (**A**) and (**B**) according to the log-rank (Mantel‒Cox) test

Multivariate analysis identified several factors independently associated with mortality among COVID-19 patients (Table 2). The presence of at least one chronic condition (AOR, 2.374; CI, 1.078–5.230; *p* = 0.0320) and older age ≥ 60 years (AOR, 6.051; CI, 2.190–16.723; *p* = 0.0010) were significantly associated with increased odds of mortality. Importantly, receiving three doses of the COVID-19 vaccine was significantly associated with lower odds of mortality (AOR, 0.229; CI, 0.108–0.486; *p* < 0.0001). Moreover, pneumonia (AOR, 6.714; CI, 4.140–10.888; *p* < 0.0001) significantly increased the odds of COVID-19 associated mortality. Kaplan‒Meier analysis additionally revealed significantly reduced survival in patients who developed pneumonia, with a 30-day survival rate of 34% compared with 60% in other COVID-19 patients (*p* < 0.0001) (Fig. 4A). Although, mechanical ventilation was not identified as an independent predictor of COVID-19 mortality, it was shown to be associated with decreased survival by Kaplan–Meier analysis (Fig. 4B). The 30-day survival rate of patients who needed mechanical ventilation was 20% compared to 79% in other COVID-19 patients (*p* < 0.0001).

**Table 2:** Independent predictors of COVID-19 mortality determined by multivariate analysis

	UOR	95% CI	p-value	AOR	95% CI	p-value
Male	1.462	1.009 to 2.122	**0.0418**			
Age < 18 years	0.098	0.042 to 0.231	** < 0.001**			
18 > Age < 60 years	0.539	0.347 to 0.833	**0.0058**			
Age ≥60 years	4.609	3.061 to 7.015	** < 0.001**	6.051	2.190 to 16.723	**0.0010**
Current smoker	0.523	0.351 to 0.774	**0.0014**			
Coinfection with Influenza A	2.002	1.100 to 3.615	**0.0191**			
Pneumonia*	6.456	4.462 to 9.342	** < 0.0010**	6.714	4.140 to 10.888	** < 0.0001**
COVID-19 vaccination						
None	1.26	0.8366 to 1.889	0.2750			
One dose	0	0.000 to 2.341	0.3860			
2 doses	0.9115	0.4904 to 1.613	0.7580			
3 doses	0.533	0.287 to 0.991	**0.0440**	0.229	0.108 to 0.486	** < 0.0001**
≥1 dose	0.7939	0.5294 to 1.195	0.2750			
≥3 doses	0.6272	0.3600 to 1.076	0.0950			
Chronic Conditions						
At least one Chronic Condition	3.502	2.170 to 5.651	** < 0.0010**	2.374	1.078 to 5.230	**0.0320**
CVD	2.429	1.688 to 3.496	** < 0.0010**			
Diabetes	1.830	1.261 to 2.658	**0.0010**			
Neoplasm	1.637	0.947 to 2.817	0.0850			
Leukemia	2.366	0.491 to 10.66	0.2550			
Renal impairment	1.467	0.800 to 2.731	0.2210			
Immunocompromised	1.355	0.832 to 2.208	0.2207			

Abbreviations: AOR, Adjusted odds ratio; CI, Confidence interval; UOR, Unadjusted odds ratio

*Pneumonia at admission or during presentation

### The genomic epidemiology of SARS-CoV-2 in Lebanon

To investigate the genomic epidemiology of SARS-CoV-2 in Lebanon, we constructed a phylogenetic tree including 138 genomes sequenced in this study and 85 Lebanese genomes retrieved from GISAID (Figure S2). We found that the prevalence of different SARS-CoV-2 clades in Lebanon has shifted markedly over time. SARS-CoV-2 clades 20A, 20B and 20C, which emerged after the original Wuhan strain, predominated between March and August 2020 (Figure S1). Among genomes collected in 2021 (*n* = 35), clade 21J (Delta) was the most prevalent (*n* = 29; 83%). The Omicron variant emerged in December 2021 with clade 21K (BA.1). Omicron became predominant by 2022, with 22B (BA.5) and 21K (BA.1) representing 59% (*n* = 37/63) and 27% (*n* = 17/63) of the sequenced genomes, respectively. Among our study samples collected between June and November 2022, clade 22B (BA.5) was dominant (89.7%, *n* = 35/39) (Figure S3). By 2023 (*n* = 74), a predominant shift toward XBB-related lineages was observed, including 22F (XBB) (*n* = 18, 24%), 23D (XBB.1.9) (*n* = 17, 23%), 23A (XBB.1.5) (*n* = 15, 20%) and 23B (XBB.1.16) (*n* = 6, 8%) (Figure S2). In 2024, only samples from our study (*n* = 38) were available for analysis, among which clade 24A (JN.1) was predominant (*n* = 19, 50%), followed by 24E (KP.3.1.1) (*n* = 8, 21%) (Figures S2 and S3).

**Fig. 5:**
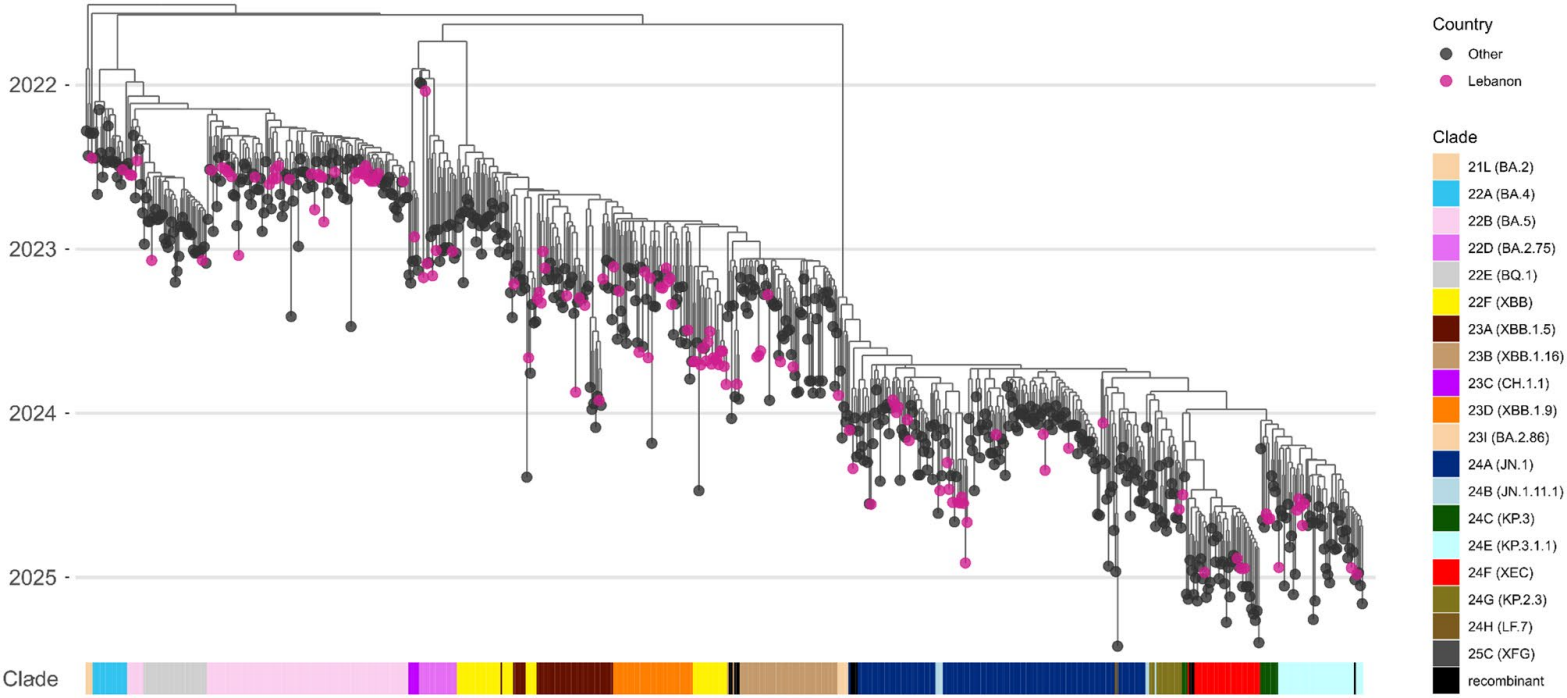
Time-resolved phylogenetic tree displaying SARS-CoV-2 genomes collected from SARI patients in Lebanon (*n* = 138) and sequences from global circulation (*n* = 567) (**A**). The tree nodes are coloured by country and the clades are differentiated by colour in the horizontal bar at the bottom of the tree

**Fig. 6:**
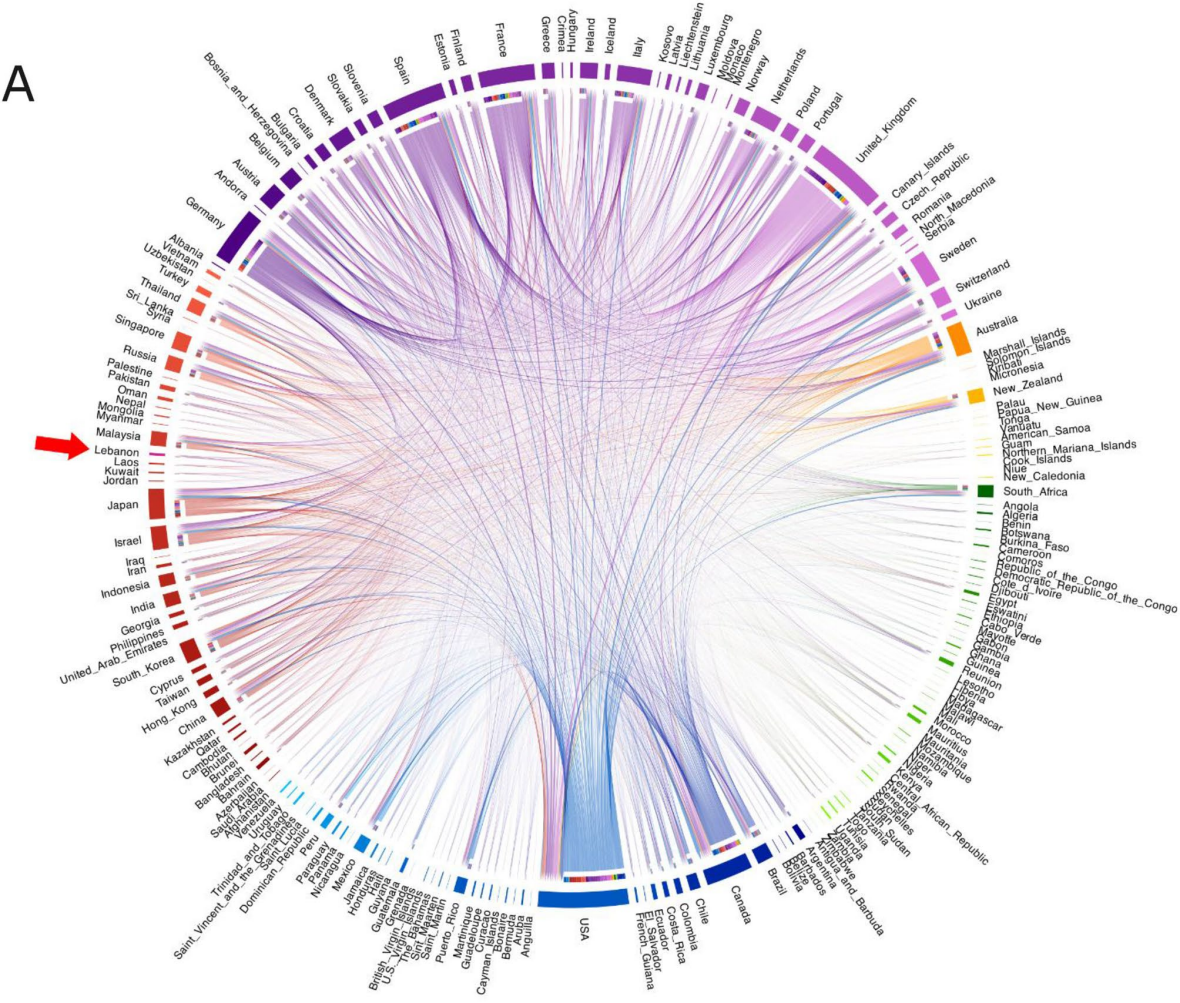

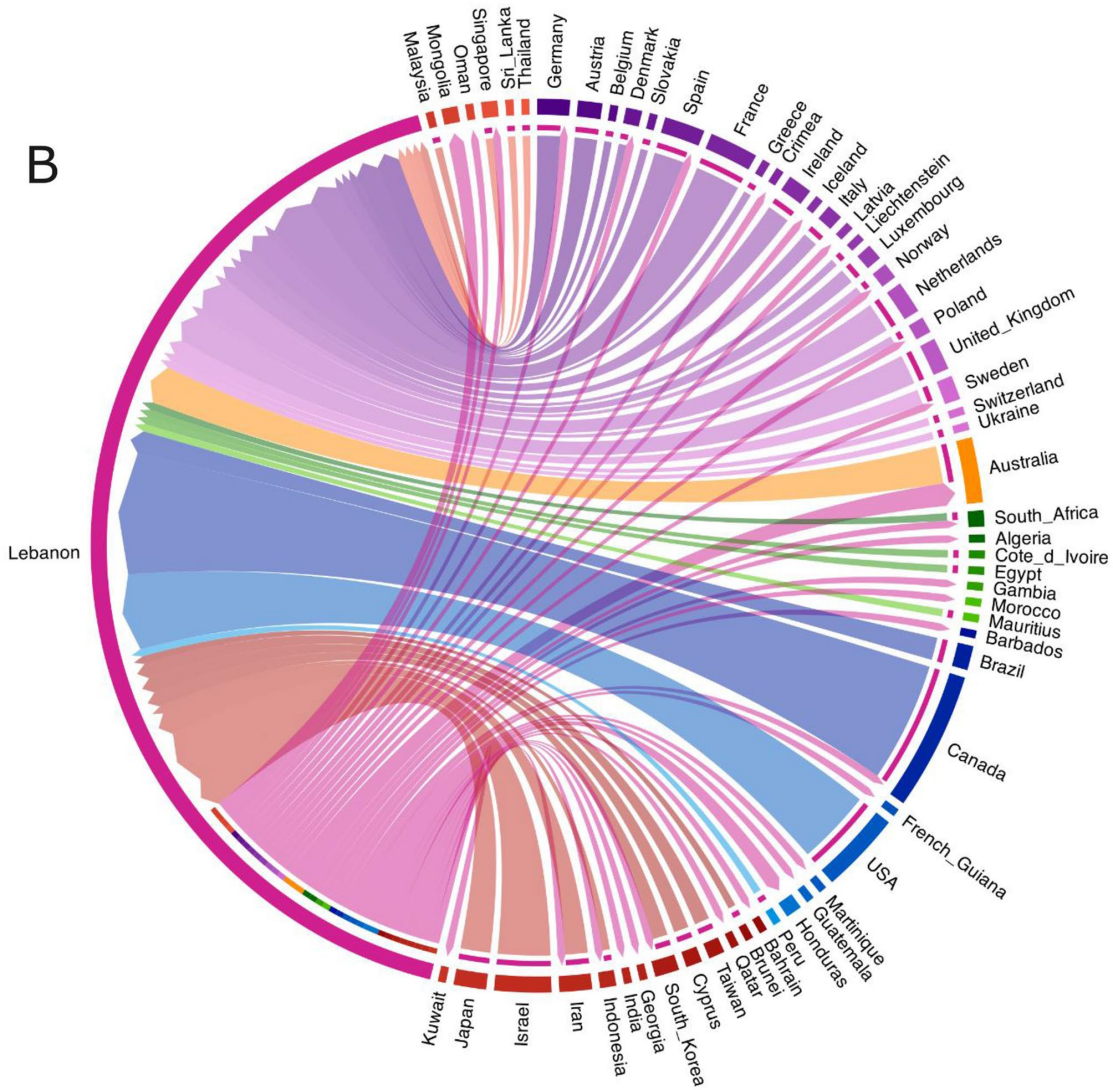
Chord diagram containing the total transitions between countries inferred from the maximum likelihood tree constructed with 138 SARS-CoV-2 genomes from Lebanon and 52,759 genomes retrieved from GISAID (**A**). Countries with inferred transitions involving Lebanon. The direction of the arrow indicates the country from which the virus was imported (**B**)

Time-resolved phylogenetic analysis revealed several distinct clusters of Lebanese genomes, including those in clades 22B (BA.5) and 22F (XBB), suggesting that more than one introduction may have led to local community transmission chains (Fig. 5). Moreover, analysis incorporating the GISAID dataset, which captures broader transmission dynamics between Lebanon and other countries, showed that introductions into Lebanon were not restricted to specific countries or continents (Fig. 6A). Countries such as Canada, the USA, Israel, France and Australia were among the sources of SARS-CoV-2 genomes most frequently observed in Lebanon (Fig. 6B). Lebanese genomes were also found alongside sequences from various other regions, which suggests potential export events. However, patterns of both importation and exportation may reflect the heterogeneity of sequencing across regions.

## Discussion

This study provides a comprehensive overview of the evolving COVID-19 landscape in Lebanon, emphasizing temporal changes in both clinical manifestations and circulating SARS-CoV-2 lineages. It documents the transition of the COVID-19 pandemic toward a flu-like epidemic starting from 2022/2023. Unlike influenza, COVID-19 did not demonstrate a clear seasonal pattern. Instead, recurrent peaks were observed across multiple years in July and August, likely driven by the influx of expatriate Lebanese returning from different regions during the summer. The transition of COVID-19 to an epidemic was marked by declining epidemic peaks over time and reduced monthly SARS-CoV-2 positivity rates among hospitalized SARI patients. During the same period, the WHO officially declared that COVID‑19 was no longer a public health emergency of international concern [30]. However, SARS-CoV-2 infections continue to pose substantial health burden and still contribute to significant clinical consequences such as the development of long coronavirus disease (COVID). The latter is characterized by persistent symptoms following SARS-CoV-2 infection and may involve autoimmune pathogenesis and organ damage [31–33].

The transition of COVID-19 from pandemic to endemic in Lebanon coincided with reduced disease severity. The most severe clinical outcomes were observed among COVID-19 patients hospitalized during 2020/2021. Although no SARS-CoV-2 genomes from that period were available for sequencing, phylogenetic analysis that included genomes retrieved from GISAID revealed that the 20A/B/C clades predominated in 2020, while the Delta variant was dominant in 2021. COVID-19 severity and mortality reached their lowest levels in 2023/2024, coinciding with the predominance of the Omicron JN.1 sublineage and its descendants. Our findings are in line with global reports demonstrating that Omicron is associated with less severe outcomes and fatalities compared to the Delta variant [34, 35]. Given this tangible impact of variants on clinical presentation, continuous context-specific evaluation of dominant SARS-CoV-2 lineages is warranted, especially in hospital settings.

The Omicron variant harbors the ORF6:D61L mutation which impairs viral immune evasion by interfering with the ability of ORF6 to antagonize interferon signaling [36]. Moreover, Omicron displays limited pathology in the lungs but replicates more efficiently in the upper respiratory tract compared to previous variants [37]. The significant reduction in the incidence of pneumonia and ARDS over the study period could be attributed to these characteristics of Omicron. However, factors such as hybrid immunity and secondary infection may have likewise played a role. Omicron is also characterized by higher levels of transmissibility [38, 39], and still causes substantial public health and patient burdens. We detected a significant increase in pediatric cases from 4% in the pre-Omicron era to 41% in 2023/2024, which emphasizes the need to strengthen public health awareness around pediatric protection through vaccination, particularly for children with comorbidities. Moreover, Omicron exhibits a distinct lung disease profile compared to earlier variants. Omicron-related pneumonia more often presents with pleural effusion and consolidations [40], which usually indicate a secondary bacterial infection. Interestingly, a significant increase in the rate of bacterial coinfections was detected in 2022/2023 and 2023/2024 (Table 1). However, these trends could be influenced by the shifts in patient demographics or treatment practices, rather than by the variant itself.

In our study, a single COVID-19 vaccine dose was associated with reduced mortality. Although this finding was not statistically significant, evidence from the literature reveals that a single dose of the Pfizer-BioNTech BNT162b2 or Oxford-AstraZeneca ChAdOx1-S vaccine can substantially reduce symptomatic COVID-19 in older adults and provide protection against severe disease [17]. Another study from the UK early in the pandemic showed that rapidly administering one vaccine dose to a larger proportion of the population was more effective at reducing COVID-19 mortality than multiple doses given to a smaller group [41]. Our study demonstrated that receiving three doses of a COVID-19 vaccine was significantly associated with lower odds of mortality in hospitalized patients. Moreover, we identified age ≥ 60 years as an independent risk factor for mortality, which is in-line with previous reports showing that older age is a risk factor for adverse COVID-19 outcomes [42–44]. Hence, booster vaccination remains essential in high-risk groups such as older adult patients. The protective effects of COVID-19 vaccination were previously documented, with studies reporting reductions in hospitalizations and ICU admissions [45, 46]. Furthermore, a study by Seo et al. revealed that vaccination significantly lowers the risk of severe disease and pneumonia among patients with SARS-CoV-2 breakthrough infections [15]. In Lebanon, two distinct vaccination campaigns were launched in February 2021 and January 2022, contributing to increased vaccine coverage over time. These vaccination efforts, likely contributed to the observed decline in disease severity and mortality in later periods of the study. As of January 2023, COVID‑19 vaccines are no longer available in Lebanon, reflecting ongoing gaps in vaccine supply and public health infrastructure that may undermine sustained protection against future outbreaks.

COVID-19 pneumonia represents a major risk factor for worse outcomes in infected patients [47, 48]. Early in the pandemic, severe COVID-19 pneumonia was shown to be associated with very high mortality, particularly in resource-constrained settings [49]. Ikenouchi et al. showed that COVID-19 pneumonia was associated with significantly higher odds of mortality, but only in patients undergoing kidney dialysis [50]. In this study, we identified chest X-ray confirmed pneumonia as an independent risk factor for mortality among all hospitalized COVID-19 patients. Moreover, we showed that COVID-19 pneumonia was associated with significantly decreased survival rates. This trend was observed across all radiologically confirmed pneumonia cases, regardless of whether mechanical ventilation was required. These findings underscore the critical importance of early detection and management of pneumonia in hospitalized COVID-19 patients and highlight the need for vigilant monitoring even in the absence of respiratory failure requiring intubation. This is particularly relevant for immunocompromised patients, who are at high risk for adverse outcomes from SARS-CoV-2–associated pneumonia and may encounter additional challenges in its clinical management [51, 52].

One of the strengths of this study is its multicenter design, with samples collected from six hospitals representing different regions across Lebanon. This enhanced the generalizability of our findings. Another notable strength is the extended duration of surveillance, which spans four years and includes a high number of SARI patients and COVID-19 cases. Additionally, the integration of SARS-CoV-2 genomic epidemiology offered valuable insights into the relationships between clinical outcomes and the dominant SARS-CoV-2 variants. Furthermore, our data sheds light on the transmission dynamics of SARS-CoV-2 in Lebanon by first examining the composition of local transmission clusters and then characterizing the profile of virus exchange between Lebanon and different regions of the world. It has already been noted that a high number of virus exchanges have taken place between Middle Eastern countries, particularly in Jordan, and those on different continents, with most of the introductions into the region in early pandemic being led by European countries and spread into the country by land travel [53].

Despite these strengths, the study has several limitations. A limited number of respiratory viruses were tested, which may have underestimated the full extent of co-infections and their association with clinical outcomes. While all the samples were tested for RSV, influenza A, influenza B and SARS-CoV-2, only a small subset was assessed using a multiplex panel targeting 21 respiratory viruses. Molecular assays remain the gold standard for viral RNA detection [54]. However, nasopharyngeal swab collection is invasive compared to alternative sampling methods [55, 56], which may have limited patient willingness to participate in the study. An additional challenge in COVID-19 diagnosis was that a subset of patients was assessed using rapid antigen tests at participating hospitals. These tests are known to exhibit lower sensitivity than molecular assays, particularly in individuals with low viral loads or outside the early phase of infection [57]. As a result, false-negative results cannot be excluded, potentially leading to underestimation of true infection rates. Diagnostic uncertainty extends beyond severe respiratory illness. Kang et al. have previously reported that infectious diseases account for the majority of cases with fever of unknown origin (FUO) [58]. However, the authors also demonstrated a significant increase in the proportion of FUO cases attributed to undiagnosed causes over the past decade, despite advances in diagnostic modalities. These findings underscore persistent gaps in infectious disease diagnosis and highlight the need for more comprehensive and sensitive approaches to improve case detection.

Moreover, data on the specific types of COVID-19 vaccines administered and the timing of the most recent dose were not consistently available. The transition from the ECDC-modified ILI case definition in 2020/2021 to the WHO Extended SARI definition in 2021/2022 and subsequent years represents a limitation that may have introduced variability in patient characteristics. Furthermore, the number of SARS-CoV-2 samples with high viral loads suitable for sequencing was relatively limited, and early-pandemic samples from 2020 to 2021 were not sequenced due to resource limitations. This may have constrained the depth of genomic analysis. While sequencing selection was based on technical feasibility, enrichment for lower-CT samples may have biased the genomic dataset toward more severe disease presentations [59].

## Conclusions

The transition of COVID-19 from pandemic to endemic in Lebanon was associated with reduced disease severity. This decrease can be attributed to changes in circulating SARS-CoV-2 variants and widespread vaccination efforts, but also to other factors such as prior infections, improved clinical management strategies, shifts in patient demographics and evolving testing practices. Despite this, vaccination remains essential, particularly in older adult patients who are at high risk of mortality. Moreover, early diagnosis and prompt management of COVID-19-related pneumonia remain critical, given its strong association with mortality. Furthermore, WGS has proven invaluable in monitoring the local evolution of SARS-CoV-2 and understanding how the changing landscape of variants impacts clinical outcomes.

## Electronic supplementary material

Below is the link to the electronic supplementary material.

Supplementary Material 1

Supplementary Material 2

Supplementary Material 3

Supplementary Material 4

## Data Availability

The datasets generated and/or analysed during the current study (*n* = 138) are available in the GISAID EpiCoV repository accessible via DOI: https://doi.org/10.55876/gis8.251120rp (GISAID Identifier: EPI_SET_251120rp). Accession numbers corresponding to the genomes generated in this study are also provided as supplementary material. All the genome sequences and associated metadata in the dataset used to construct the time-resolved phylogeny (Fig. 5) are published in GISAID’s EpiCoV database (GISAID Identifier: EPI_SET_250819me) (DOI: https://doi.org/10.55876/gis8.250819me). An interactive visualization of the phylogeny constructed with local genomes from Lebanon (Figure S1) is available in microreact (https://microreact.org/project/7HaDEnmHv1murAQFTroCpH-lebanonclades).

## Abbreviations

AOR: Adjusted Odds ratios
ARDS: Acute respiratory distress syndrome
CIDR: Center for Infectious Diseases Research
CI: Confidence interval
COVID-19: Coronavirus disease 2019
CVD: Cardiovascular disease
CT: Cycle threshold
ECDC: European Centre for Disease Prevention and Control
FUO: Fever of unknown origin
GIHSN: Global Influenza Hospital Surveillance Network
ICU: Intensive care unit
ILI: Influenza-like illness
IQR: Interquartile range
MENA: Middle East and North Africa
QC: Quality control
RSV: Respiratory syncytial virus
SARI: Severe acute respiratory infection
SARS-CoV-2: Severe acute respiratory syndrome coronavirus 2
UOR: Unadjusted odds ratio
WGS: Whole genome sequencing
WHO: World Health Organization.
