# Supplementary material for: Genomic and clinical epidemiology of SARS-CoV-2 in Lebanon: a prospective multicenter study 2020–2024
Source: BMC Infect Dis. 2026 Jan 28;26:424. doi: 10.1186/s12879-026-12635-w (PMC12924613; doi:10.1186/s12879-026-12635-w)
Supplement: Supplementary file 2 — Supplementary Material 2 [file 12879_2026_12635_MOESM2_ESM.pdf]

Subject Study Number: ……………

Global Influenza Hospital Surveillance Network

Any **positive** respiratory virus obtained at the hospital:

……………

Core questionnaire: Patients of all ages

Version 7.5 *11 October 2023*

Screening

1) **Does the patient comply with any of the admission diagnosis listed in Annex 1?**
   0|__|No 1|__| Yes
   a. **Admission diagnosis** (letter/code.subcode) |__|__|__|__|.|__|__|
   b. **ICD used** 9☐ ICD-9 10☐ ICD-10
2) **Date of admission** (dd-mm-yyyy) |__|__| - |__|__|- |__|__|__|__|
3) **Date of questionnaire** (dd-mm-yyyy) |__|__| - |__|__|- |__|__|__|__|
4) **Patient study identification number** |__|__|__|__|__|__|__|__|__|
5) **Sex:** 1☐ Male 2☐ Female
6) **Age** |__|__|__| O days O months O years
7) **Place of residence: ……………………………………………..**

Signs and symptoms

8) **Has the patient had any one of these symptoms in the last 7 -10 days prior to admission?**

a. **ILI systemic symptoms**

Fever/Feverishness	0|__|No	1|__| Yes	
Malaise/fatigue/lethargy	0|__|No	1|__| Yes	
Headache	0|__|No	1|__| Yes	2|__| NA
Myalgia/muscle ache/body ache	0|__|No	1|__| Yes	2|__| NA

b. **ILI respiratory symptoms**

Cough	0|__|No	1|__| Yes	
Sore throat	0|__|No	1|__| Yes	2|__| NA
Shortness of breath/difficult breathing	0|__|No	1|__| Yes	
Wheezing	0|__|No	1|__| Yes	
Nasal congestion/runny nose	0|__|No	1|__| Yes	

c. Date of onset of symptoms (dd-mm-yyyy): ……./……./………

American University of Beirut
Institutional Review Board
08 November 2023
APPROVED

9) Case definition: Extended SARI case definition: An acute respiratory infection with cough and onset within 10 days that requires hospitalization (no fever required).

Does the patient case comply with this case definition? 0|__|No 1|__| Yes

If yes, please continue the questionnaire. If no, then the questionnaire should be closed here.

10) Other signs or symptoms **at disease presentation (i.e. in the past 10 days), mark all that apply**:

a. Nausea or Vomiting	0|__|No 1|__| Yes
b. Diarrhea	0|__|No 1|__| Yes
c. New loss of taste or smell	0|__|No 1|__| Yes 2|__| NA
d. Chest pain	0|__|No 1|__| Yes 2|__| NA
e. Others	________________

Swabbing

11) PCR Sample source taken from GIHSN team:

Nasal	0☐No	1☐Yes
Nasopharyngeal	0☐No	1☐Yes
Pharyngeal	0☐No	1☐Yes

Date of swabbing (dd-mm-yyyy)? |__|__| - |__|__|- |__|__|__|__|

OR/AND

PCR Sample taken from the laboratory of the hospital? 0☐No 1☐Yes, result:……..

If yes, Nasal	0☐No	1☐Yes
Nasopharyngeal	0☐No	1☐Yes
Pharyngeal	0☐No	1☐Yes

Date of sampling (dd-mm-yyyy)? |__|__| - |__|__|- |__|__|__|__|

Was a respiratory panel performed? 0☐No 1☐Yes, result:……..

OR/AND

Rapid Antigen for any respiratory virus was done at the hospital? 0☐No 1☐Yes, result:………………………..

Date of sampling (dd-mm-yyyy)? |__|__| - |__|__|- |__|__|__|__|

OR/AND

If PCR OR Rapid Antigen was done outside the hospital before admission (for patients with positive Respiratory virus ONLY): 0☐No 1☐Yes, result:……………..

Date of sampling (dd-mm-yyyy)? |__|__| - |__|__|- |__|__|__|__|

American University of Beirut
Institutional Review Board
08 November 2023
APPROVED

Laboratory Results

12) **a. Does the patient have a positive flu result?** 0☐ No 1☐ Yes 2☐ Inadequate sample

b. If yes, tick the boxes corresponding to the positive virus(es)

- H1N1pdm09 0☐ No 1☐ Yes
- H3N2 0☐ No 1☐ Yes
- B/Yamagata 0☐ No 1☐ Yes
- B/Victoria 0☐ No 1☐ Yes
- Influenza A no lineage 0☐ No 1☐ Yes
- Influenza B no lineage 0☐ No 1☐ Yes

13) **a. Did you test for other respiratory viruses (*optional*)?**
0☐ No 1☐ Yes 2☐ Inadequate sample

b. If yes, tick the boxes corresponding to the positive virus(es)

- SARS-CoV-2 0☐ No 1☐ Yes
- Human Corona virus 0☐ No 1☐ Yes
- Metapneumovirus 0☐ No 1☐ Yes
- Respiratory syncytial virus 0☐ No 1☐ Yes
- Adenovirus 0☐ No 1☐ Yes
- Bocavirus 0☐ No 1☐ Yes
- Parainfluenza virus 0☐ No 1☐ Yes
- Rhinovirus 0☐ No 1☐ Yes
- Others, specify:…… 0☐ No 1☐ Yes

14) **Have you detected a co-infection?** **0**☐ No 1☐ Yes 2☐ Inadequate sample

Patient characteristics

15) **Household crowding index (HCI):**

How many rooms (without kitchen and bathrooms) is the patient's house composed of?
………………………

How many people (other than newborns) live in this house (Patient's house)?
………………………………….

HCI: Number of co-residents per household (excluding newborn infants)/ the total number of rooms, excluding the kitchen and bathrooms =

≤ 1 less than 1 person per room reflected a less crowded house so high socio-economic level
> 1 more than one person per room reflecting a house too crowded so low socio-economic level.

American University of Beirut
Institutional Review Board
08 November 2023
APPROVED

16) What is the educational level of the patient or the mother/caregiver if patient aged <5 years old?

0|__|No Education
1|__| Primary Education
2|__| Secondary Education
3|__| Higher Education

17) Occupation/social class (see list below) of the patient or the father or mother of the patient if patient is <5years old |__|

1. Executives of the public administration and companies with 10 or more employees. Professions with a graduate (MD, lawyers, architects) or a Post graduate degree (PhD).
2. Managers of firms with fewer than 10 employees. Professions associated with a first cycle university degree (diploma). Technicians. Artists. Athletes.
3. Administrative employees and professionals that give administrative support or financial management (accountants). Personal services (i.e. Hairstylist). Security. Self-employed. Supervisors.
4. Skilled manual workers (Specific training needed).
5. Semiskilled manual workers (No specific training but working on specific general fields giving assistance to the skilled manual workers: industry, building, furniture, and fishing).
6. Unskilled workers.
7. Not classifiable/don't know.

18) Works at a health care facility or setting? 0☐ No 1☐ Yes

19) Smoking habits of the patient or their parents/tutors if patient's age <14 years old

For patients between 5 and 13 years old, the answer will refer to the smoking habits of the parents/tutors. Please, mark either the option 'current smoker' or the option 'past smoker' if any of the parents/tutors is a current or a past smoker, considering the most recent situation of the patient and any of the parents/tutors.

For patients 14 years old or more, please mark the most adequate option that describes the smoking habits of the patient.

1|__| Current smoker
2|__| Past smoker
3|__| Never smoker

American University of Beirut
Institutional Review Board
08 November 2023
APPROVED

Clinical history

20) **Height** (*Round up to the nearest integer*) |__|__|__|

Weight (*Round up to the nearest integer*) |__|__|__|

21) **Pregnancy status:** **0**☐ No 1☐ Yes 2☐ NA

Note: Pregnancy status is NA if patient is male OR patient is female but her age not within the childbearing age (12-51 years old)

If yes, pregnancy weeks: |__|__|__| |__|Don't know

22) If the patients is a child aged < 5 years old, is the child being breast-fed?

0☐ No 1☐ Yes 2☐ NA

If yes, how many months were the child breastfed? 1|__| Less than 3 months

2|__| 3 to less than 6 months

3|__| 6 months or more

4|__| Baby is less than 3 months and breastfeeding is still ongoing

23) **a. Does the patient have any chronic conditions?** 0☐No 1☐ Yes

b. If yes, indicate which ones

Cardiovascular disease / High blood pressure	0☐No	1☐ Yes
Chronic obstructive pulmonary disease	0☐No	1☐ Yes
Asthma	0☐No	1☐ Yes
Diabetes	0☐No	1☐ Yes
Immunodeficiency (except HIV)/Organ transplant	0☐No	1☐ Yes
Renal impairment	0☐No	1☐ Yes
Rheumatologic disease / Autoimmune disease	0☐No	1☐ Yes
Neurological or neuromuscular disease	0☐No	1☐ Yes
Cirrhosis / Liver disease	0☐No	1☐ Yes
Neoplasm (active)	0☐No	1☐ Yes
Obesity	0☐No	1☐ Yes
Malnutrition (Only for children < 5 years)	0☐No	1☐ Yes
Active tuberculosis	0☐No	1☐ Yes
HIV infection	0☐No	1☐ Yes
HIV exposure (if children < 5 year)	0☐No	1☐ Yes
Leukemia	0☐No	1☐ Yes
Hemoglobinopathy	0☐No	1☐ Yes
Born premature, ie., <37 week gestation (Only for children < 5 years)	0☐No	1☐ Yes
Other, specify:…………………	0☐No	1☐ Yes

American University of Beirut
Institutional Review Board
08 November 2023
APPROVED

24) a. **Use of influenza antivirals (**oseltamivir, zanamivir, favipiravir or peramivir) **for the current episode initiated before this admission?**

0☐ No 1 ☐ Yes 2 ☐ Do not know

b. **Starting Date** (dd-mm-yyyy) |__|__| - |__|__|- |__|__|__|__|

c. Use of other antivirals preceding admission? 0☐ No 1 ☐ Yes 2 ☐ Do not know

d. Starting Date (dd-mm-yyyy) |__|__| - |__|__|- |__|__|__|__|

25) a. Use of influenza antiviral (oseltamivir, zanamivir, favipiravir o peramivir) for the current episode initiated during this admission?

0☐ No 1 ☐ Yes 2 ☐ Do not know

b. Starting Date (dd-mm-yyyy) |__|__| - |__|__|- |__|__|__|__|

c. Use of other antivirals during this admission? 0☐ No 1 ☐ Yes 2 ☐ Do not know

d. Starting Date (dd-mm-yyyy) |__|__| - |__|__|- |__|__|__|__|

26) a. Use of antibiotics preceding this admission?

0☐ No 1 ☐ Yes 2 ☐ Do not know

b. Starting Date (dd-mm-yyyy) |__|__| - |__|__|- |__|__|__|__|

27) a. Use of antibiotics during this admission?

0☐ No 1 ☐ Yes 2 ☐ Do not know

b. Starting Date (dd-mm-yyyy) |__|__| - |__|__|-|__|__|__|__|

28) Bacterial co-infection (Lab proven): 0☐No 1☐Yes 2☐ Do not know

Vaccination Status

29) <u>Influenza Vaccination:</u>

a. **Influenza vaccination for the <u>current season</u>** 0☐ No 1☐ Yes 2☐ Do not know

b. Date of last influenza vaccine dose (dd-mm-yyyy)|__|__| |__|__| |__|__|__|__|

☐ Do not know

c. **Vaccinated more than 14 days before onset of the acute respiratory symptoms?** 0☐ No 1☐ Yes 2☐ Do not know

American University of Beirut
Institutional Review Board
08 November 2023
APPROVED

d. Vaccinated within 6 months before onset of the acute respiratory symptoms? 0□No 1□Yes 2□ Do not know

e. Vaccination history for current season validated through registry or medical records? 0□No 1□Yes 2□ Do not know

f. Type of influenza vaccine? □IIV3 □IIV4 □Do not know

g. Influenza vaccination in the preceding season? 0□ No 1□ Yes 2□ Do not know

<u>COVID-19 Vaccination:</u>

a. How many COVID-19 vaccine doses received? O None O One O Two O Three O Four O Five or more O Do not know

b. Date of last vaccine dose (dd-mm-yyyy) |__| - |__|__| - |__|__|__|__| O Do not know

c. If at least one dose received, which type(s) of COVID-19 vaccine(s)? *(check all that apply)*

1O mRNA (e.g., Moderna, Pfizer BioNtech)

2O Inactivated whole virion (e.g., Sinopharm, Sinovac)

3O Viral vector vaccine (e.g., AstraZenica, Sputnik)

4O Protein subunit vaccine (e.g., Novavax)

5O Do not know

Exposure

30) Sick contacts: 0□No 1□Yes 2□ Do not know

If yes, 1|__| Household members 2|__| school/daycare 3|__| workplace

31) History of Travel (within 14 days): 0□No 1□Yes 2□ Do not know

If yes, city?..............

Severity (measured at admission)

32) Confusion/lethargy 0□ No 1□ Yes 2□ Do not know

33) Blood pressure (systolic/diastolic) |__|__|__| / |__|__|__|mmHg □ Do not know

American University of Beirut
Institutional Review Board
08 November 2023
APPROVED

34) **Respiratory rate at admission (breaths per minute)** |__|__|bpm ☐ Do not know

35) **Oxygen saturation value on ambient air:** |__|__|__|%

36) **Supplemental oxygen without mechanical ventilation** 0☐ No 1☐Yes 2☐ Do not know

37) **Vasopressor support** 0☐ No 1☐Yes 2☐ Do not know

38) **Apnea (if the patient is a child < 5 years old)** 0☐ No 1☐Yes 2☐ Do not know

39) What is the baseline frailty score of the patient? **(for all patients 50 years and older), prior to onset of the current illness**? (category 1-9) (see annex 2 for definition of the scale) Category |__|__| ☐ Did not ask (if NA) ☐ Do not know

Severity (measured at any time during admission)

40) ICU admission
If yes, duration of ICU stay----------------------------- 0☐ No 1☐ Yes 2☐ Do not know

41) High dependence unit (at any time during hospitalization) 0☐ No 1☐ Yes 2☐ Do not know

42) Invasive Mechanical ventilation (Intubation) 0☐ No 1☐ Yes 2☐ Do not know

43) Complications during hospital stay:
0☐ No 1☐Yes 2☐ Do not know

1|__| Pneumonia (Chest X-Ray confirmed)
2|__| Acute Respiratory Disease Syndrome (ARDS)
3|__| Croup
4|__|Encephalopathy/encephalitis
5|__| Shock
6|__|Guillain-Barre syndrome
7|__| Bronchiolitis
8|__|Hemorrhagic pneumonia/pneumonitis
9|__| Sepsis
10|__| Reye syndrome
11|__| Stroke
12|__| Renal failure
13|__|Seizures
14|__|Pericarditis
15|__| Cardiomyopathy/myocarditis
16|__| MI
17|__| Liver failure
18|__| UTI
19|__| Myositis
20|__|Other:………..

Outcome

44) Death while hospitalized 0☐ No 1☐ Yes 2☐ Do not know

45) Discharge/death date (dd-mm-yyyy) |__|__| - |__|__| - |__|__|__|__|

46) Transfer to another hospital/Left against medical orders 0☐ No 1☐ Yes 2☐ Do not know

American University of Beirut
Institutional Review Board
08 November 2023
APPROVED

47) a. Main diagnose at discharge/death |__|__|__| . |__|__|
(letter/code.subcode**)**

b. Secondary 1 diagnose at discharge/death |__|__|__| . |__|__|
(letter/code.subcode**)**

c. Secondary 2 diagnose at discharge/death |__|__|__| . |__|__|
(letter/code.subcode**)**

48) What is the frailty score of the **patient (for all patients 50 years and older) at discharge**? (category 1-9) (see annex 2 for definition of the scale) Category |__|__| ☐ Did not ask (if NA)

☐ Do not know

49) Duration of hospital stay (in days): ……………

End of the questionnaire. Please send the questionnaire to PI for recording.

American University of Beirut
Institutional Review Board
08 November 2023
APPROVED

Annex 1: Admission diagnosis

Case ascertainment/Case finding

You can use this Table as guidance to identify patients that may be eligible to participate in the surveillance system. You can use the list of acute events and/or ICD codes if available at your hospital or you can rely on other case ascertainment strategies, like looking at hospital admission logs, or looking at emergency department logs, infectious disease contacts etc.

Table 1. Example of admission diagnoses possibly associated with an influenza infection that could be taken into account when looking for eligible patients.
International Classification of Diseases Code version 9 and 10.

For patients less than 5 years	ICD 9 Codes	ICD 10 Codes
Acute upper or lower respiratory disease	382.9; 460 to 466	J00-J06, J20-J22
Dyspnea, breathing anomaly, shortness of breath, tachypnea (polypnea)	786.0; 786.00; 786.05-786.07; 786.09; 786.9	R06.0, R06, R06.9, R06.3, R06.00, R06.09, R06.83, R06.02, R06.82, R06.2, R06.89
Acute asthma or exacerbation	493.92	J45.901
Pneumonia and influenza	480 to 488	J09-J18
Acute respiratory failure	518.82	J96
Acute heart failure	428-429.0	I50-I50.9; I51.4
Myalgia	729.1	M79.1
Altered consciousness, convulsions, febrile convulsions	780.01-780.02; 780.09; 780.31-780.32	R40.20, R40.4, R40.0, R40.1, R56.00, R56.01
Fever or fever unknown origin or non specified	780.6-780.60	R50, R50.9
Cough	786.2	R05
Gastrointestinal manifestations	009.0; 009.3	A09.0; A09.9
Sepsis, Systemic inflammatory response syndrome, not otherwise specified	995.90-995.94	R65.10, R65.11, R65.20, A41.9

American University of Beirut
Institutional Review Board
08 November 2023
APPROVED

Nausea and vomiting	078.82; 787.0; 787.01-787.03	R11; R11.0; R11.10 - R11.12; R11.2
Loss of smell, loss of taste		R43.8 , R43.8,
Pneumonia due to coronavirus disease 2019		J12.82, U07.1,
Coronavirus infection, unspecified		B34.2, U07.1, J12.81
SARS-associated coronavirus as the cause of diseases classified elsewhere		B97.21

For patients 5 years and older	ICD 9 Codes	ICD 10 Codes
Acute upper or lower respiratory disease	382.9; 460-466	J00-J06, J20-J22, H66.90
Acute myocardial infarction or acute coronary syndrome	410-411 and 413-414	I20-I25.9
Acute asthma or exacerbation	493.92	J45.901
Acute Heart failure	428-429.0	I50-I50.9; I51.4
Pneumonia and influenza	480-488	J09-J18
Bronchitis and exacerbations of Chronic Pulmonary Obstructive disease	490, 491.21 and 491.22,	J40; J44.0; J44.1
Acute respiratory failure	518.82	J96
Myalgia	729.1	M79.1
Acute metabolic failure (diabetic coma, renal dysfunction, acid-base disturbances, alterations to the water balance)	250.1- 250.3; 584-586; 276-277	E11.9, E10.9, E11.65, E10.65, E10.11, E11.01, E10.641, E11.641, E10.69, E11.00, E10.10, E11.69, N17.0, N17.1, N17.2, N17.8, N17.9, N18.1, N18.2, N18.3, N18.4, N18.5,

American University of Beirut
Institutional Review Board
08 November 2023
APPROVED

		N18.6M N18.9, N19, E87.0, E87.1, E87.2, E87.3, E87.4, E87.5, E87.6, E87.70, E87.71, E87.79, E86.0, E86.1
Altered consciousness, convulsions, febrile convulsions, syncope and collapse	780.01-780.02; 780.09; 780.2; 780.31-780.32	R40.20, R40.4, R40.0, R40.1, R55, R56.00, R56.01
Dyspnea/respiratory abnormality	786.0	R06.0, R06-R06.9
Respiratory abnormality	786.00	R06.9
Shortness of breath	786.05	R06.02
Respiratory abnormality not otherwise specified	786.09	R06.3, R06.00, R06.09, R06.83
Respiratory symptoms/chest symptoms	786.9	R06.89
Fever or fever unknown origin or non-specified	780.6-780.60	R50, R50.9
Cough	786.2	R05
Sepsis, Systemic inflammatory response syndrome	995.90-995.94	R65.10, R65.11, R65.20, A41.9
Loss of smell, loss of taste		R43.8 , R43.8,
Pneumonia due to coronavirus disease 2019		J12.82, U07.1,
Coronavirus infection, unspecified		B34.2, U07.1, J12.81
SARS-associated coronavirus as the cause of diseases classified elsewhere		B97.21

American University of Beirut
Institutional Review Board
08 November 2023
APPROVED

Annex 2: Frailty scale

The frailty scale according to the categories defined below. If a subject is in between levels use best judgement.

Category 1: Very Fit. People who are robust, active, energetic and motivated. The people commonly exercise regularly. They are among the fittest for their age.

Category 2: Well. People who have no active disease symptoms but are less fit than category 1. Often, they exercise or are very active occasionally, e.g. seasonally

Category 3: Managing Well. People whose medical problems are well controlled but are not regularly active beyond routine walking.

Category 4: Vulnerable. While not dependent on others for daily help, often symptoms limit activities. A common complaint is being "slowed up", and/or being tired during the day.

Category 5: Mildly Frail. These people often have more evident slowing, and need help in high order IADLs (finances, transportation, heavy housework, medications). Typically, mild frailty progressively impairs shopping and walking outside alone, meal preparation and housework.

Category 6: Moderately Frail. People need help with all outside activities and with keeping house. Inside, they often have problems with stairs and need help with bathing and might need minimal assistance (cuing, standby) with dressing.

Category 7: Severely Frail. Completely dependent for personal care, from whatever cause (physical or cognitive). Even so, they seem stable and not at high risk of dying (within ~ 6 months)

Category 8: Very Severely Frail. Completely dependent, approaching the end of life. Typically, they could not recover even from a minor illness.

Category 9: Terminally Ill. Approaching the end of life. This category applies to people with a life expectancy <6 months, who are not otherwise evidently frail.

American University of Beirut
Institutional Review Board
08 November 2023
APPROVED
