# Supplementary material for: Genomic and clinical epidemiology of SARS-CoV-2 in Lebanon: a prospective multicenter study 2020–2024
Source: BMC Infect Dis. 2026 Jan 28;26:424. doi: 10.1186/s12879-026-12635-w (PMC12924613; doi:10.1186/s12879-026-12635-w)
Supplement: Supplementary file 4 — Supplementary Material 4 [file 12879_2026_12635_MOESM4_ESM.docx]

**Genomic and Clinical Epidemiology of SARS-CoV-2 in Lebanon: A Prospective Multicenter Study 2020-2024**

**Supplementary Materials**

**Table S1: An overview of the study sites, including location and catchment area.**

| **Hospital name** | **Hospital Information** | **Population/catchment area** | **District** |
| --- | --- | --- | --- |
| **American University of Beirut Medical Center (AUBMC)** | Urban Academic/Tertiary 373 patient-beds | All age groups/2.4 million | Beirut |
| **Rafic Hariri University Hospital (RHUH)** | Urban Academic/General 430 patient-beds | All age groups/2.4 million | Beirut |
| **Keserwan Medical Center (KMC)** | Urban Non-Academic/General 65 patient-beds | All age groups/100,000 | Northern Mount Lebanon |
| **Bekaa Hospital (BH)** | Rural Academic/General 154 patient-beds | All age groups/200,000 | Bekaa |
| **New Mazloum Hospital (NMH)** | Urban Non-Academic/General 100 patient-beds | All age groups/500,000 | North Lebanon |
| **Hammoud Hospital University Medical Center (HHUMC)** | Urban Academic/Tertiary 325 patient-beds | All age groups/250,000 | South Lebanon |

**Table S2: Demographic and clinical characteristics of 7,708 hospitalized SARI patients stratified by SARS-CoV-2 infection.**

|  | **Other patients with SARI (n=6,406)** | **COVID-19 patients (n=1302)** | **Total (n=7,708)** | **p-value** |
| --- | --- | --- | --- | --- |
| **Clinical and Demographic Characteristics** |  |  |  |  |
| **Male, n (%)** | 3,646/6,403 (57%) | 753 (58%) | 4,399/7,705 (57%) | 0.5533 |
| **Age < 18 years, n (%)** | 3874/6,403 (61%) | 319 (25%) | 4,193/7,705 (54%) | **<0.0001** |
| **18 > Age < 60 years, n (%)** | 857/6,403 (13%) | 368 (28%) | 1,225/7,705 (16%) | **<0.0001** |
| **Age > 60 years n (%)** | 1672/6,403 (26%) | 615 (47%) | 2,287/7,705 (30%) | **<0.0001** |
| **COVID-19 Vaccination** | 1,670/5,833 (29%) | 457/1,130 (40%) | 2,127/6,963 (30.5%) | **<0.0001** |
| **Comorbidities, n (%)** |  |  |  |  |
| **Chronic Conditions** | 2,870/6,401 (45%) | 840/1,301 (65%) | 3,710/7,702 (48%) | **<0.0001** |
| **CVD** | 1,737/6,401 (27%) | 589/1,301 (45%) | 2,326/7,702 (30%) | **<0.0001** |
| **Diabetes** | 878/6,399 (14%) | 312/1,301 (24%) | 1,190/7,700 (15%) | **<0.0001** |
| **Neoplasm** | 233/6,401 (4%) | 100/1,301 (8%) | 333/7,702 (4%) | **<0.0001** |
| **Leukemia** | 48/6,399 (1%) | 10/1301 (1%) | 58/7700 (1%) | 0.9438 |
| **Immunocompromised** | 250/4,494 (6%) | 107/1,028 (10%) | 357/5,522 (6%) | **0.0004** |
| **Outcomes, n (%)** |  |  |  |  |
| **ICU Admission** | 1,221/6,402 (19%) | 352/1,301 (27%) | 1,573/7,703 (20%) | **<0.0001** |
| **Oxygen Supplementation** | 1,956/6,341 (31%) | 609/1,259 (48%) | 2,565/7,600 (34%) | **<0.0001** |
| **Vasopressor** | 129/6,364 (2%) | 98/1,295 (8%) | 227/7,659 (3%) | **<0.0001** |
| **Mechanical Ventilation** | 233/6,394 (3%) | 134/1,296 (10%) | 367/7,690 (5%) | **<0.0001** |
| **Complications** | 920/6,302 (15%) | 335/1,282 (26%) | 1,255/7,584 (16%) | **<0.0001** |
| **Decreased Po Intake** | 588/4,495 (13%) | 125/1028 (12%) | 713/5,523 (13%) | 0.4266 |
| **Viral Co-infections** | 129 (2%) | 124 (9.5%) | 253 (3%) | **<0.0001** |
| **Bacterial Co-infections** | 1,772/5,812 (30%) | 281/1,136 (25%) | 2,053/6,948 (29%) | **0.0001** |
| **Sepsis** | 126 (2%) | 91 (7%) | 217 (3%) | **<0.0001** |
| **ARDS** | 110 (2%) | 107 (8%) | 217 (3%) | **<0.0001** |
| **Chest X-ray Confirmed Pneumonia** | 432 (7%) | 184 (14%) | 616 (8%) | **<0.0001** |
| **Mortality** | 222/6,400 (3%) | 141/1,299 (11%) | 363/7,769 (5%) | **<0.0001** |

**
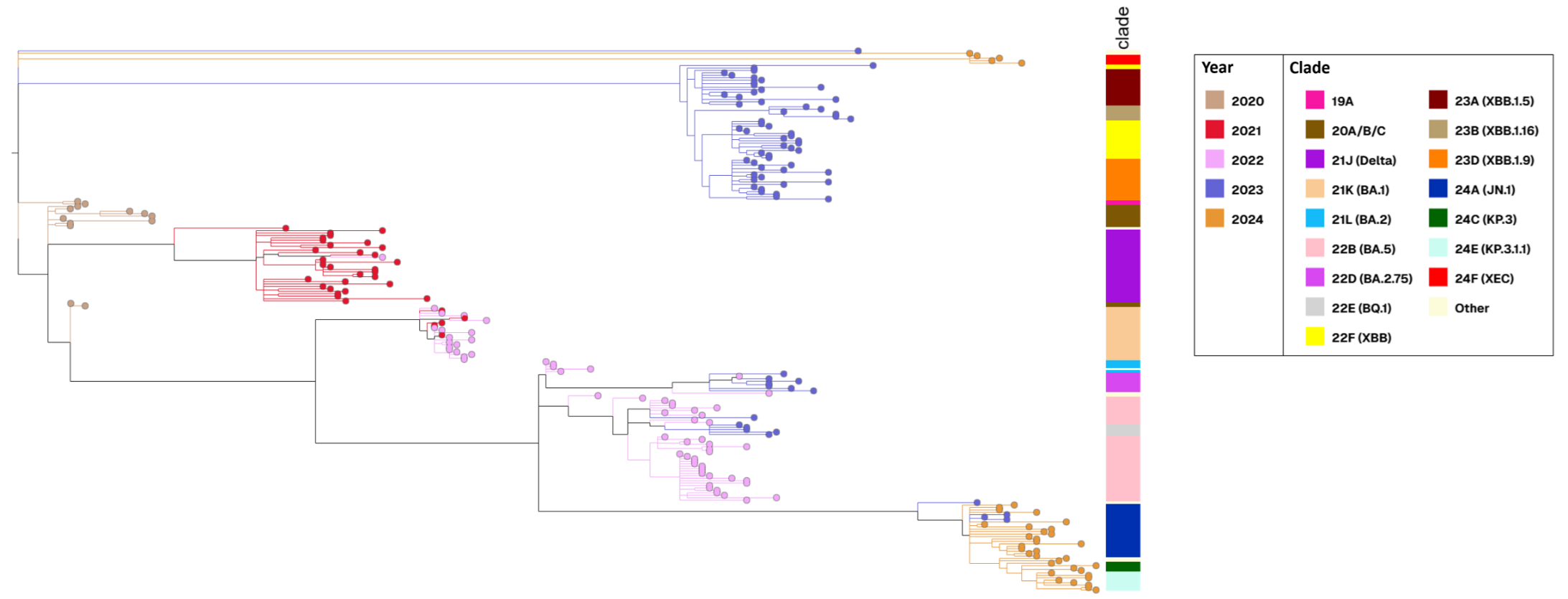
**

**Figure S1: Phylogenetic tree of SARS-CoV-2 isolates** **collected from hospitalized SARI patients in Lebanon (n=138).** The tree additionally includes 85 genomes from Lebanon retrieved from GISAID. The tree nodes are coloured by year.

Interactive phylogeny along with country and patient metadata can be visualized at https://microreact.org/project/7HaDEnmHv1murAQFTroCpH-lebanonclades

Recombinant viruses (n=2) and clades with count < 2 were designated as “Other” in the figure. These included: 23C (CH.1.1) (n=1), 21L (BA.2) (n=1), 22A (BA.4) (n=1), 23I (BA.2.86) (n=1) and 21I (Delta) (n=1), 24B (JN.1.11.1) (n=1) and 24G (KP.2.3) (n=1).

**
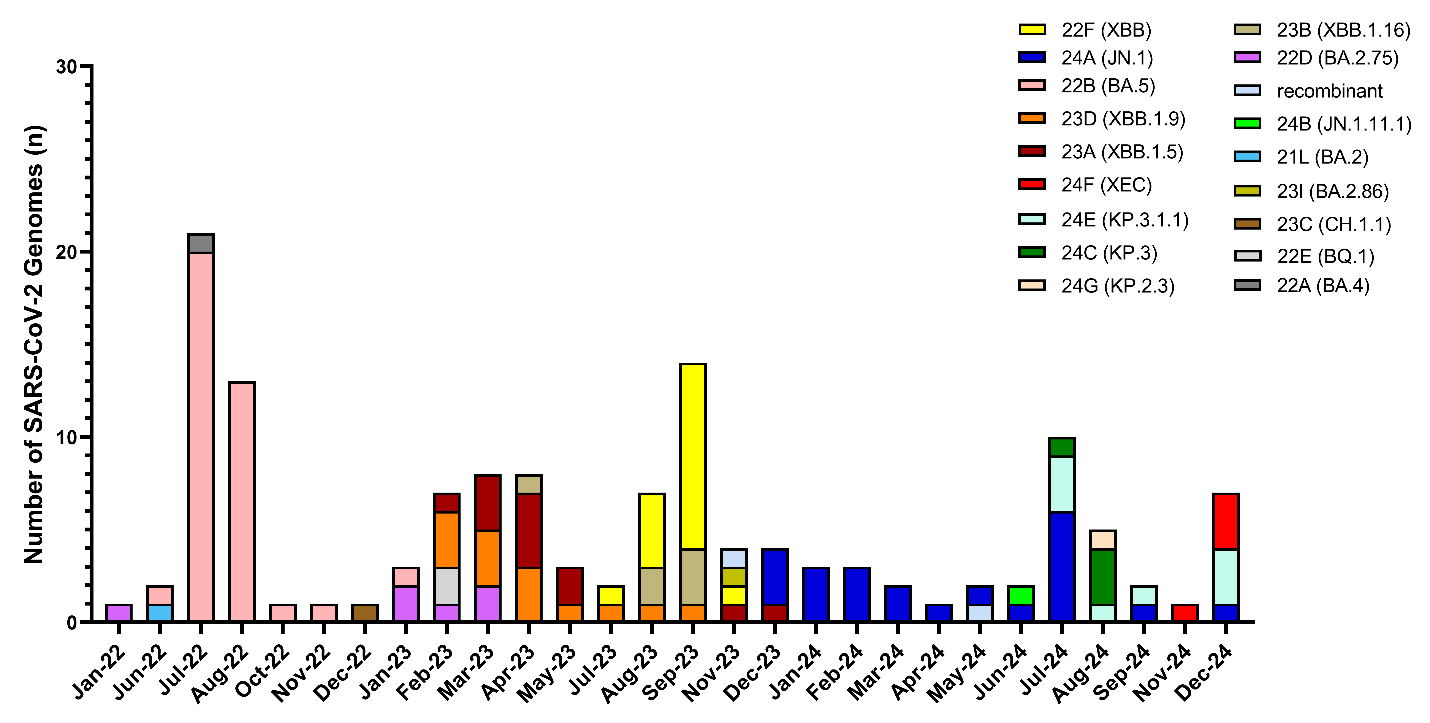
**

**Figure S2: Monthly distribution of SARS-CoV-2 clades and lineages among hospitalized SARI patients in Lebanon (n=138).**
